# Supplementary material for: Chromothripsis during telomere crisis is independent of NHEJ, and consistent with a replicative origin
Source: Genome Res. 2019 May;29(5):737–49. doi: 10.1101/gr.240705.118 (PMC6499312; doi:10.1101/gr.240705.118)
Supplement: Supplemental Material [file supp_gr.240705.118_Supplemental_file_1.zip › contigs/annotated_contigs/DB94/contig.2.DB94_length_306_mean_cov_30.0261437908.docx]

**DB94_length_306_mean_cov_30.0261437908**

```
TTATCAGCTTATCTCTGTTTTATAACTGTATTTTAATTAGTGTTATACATAAATTTGTTATATTATTTTGAAAAAAGCTTATGGAGGGA
 >chr9:102849294-102849550 - E=4e-137 p=2e-03
GTTGTGTTTTGTCGTATAGTCCATTGGTTTTAGAATTAAAGGAGATTAGGGTTTGAATTCTGTTCTGCCATTAATTTGGACACGTCACT

TAGCTTATTTGAATCTTATTCGTTTAATAAATATTCTCCCTTTTACTGTATGCCAGAGACTGCAAAGTTTTTTTT|TTT|TTTTTTTTT
                                                                            >chr16:7044
TTTTTTTTTTTTTTTTTTTTTTTTTTTTTTTTTTTTGTTT|T2789-70442841 - E=7e-14
```
